# Performance of Large Language Models ChatGPT and Gemini on Workplace Management Questions in Radiology

**DOI:** 10.3390/diagnostics15040497

**Published:** 2025-02-19

**Authors:** Patricia Leutz-Schmidt, Viktoria Palm, René Michael Mathy, Martin Grözinger, Hans-Ulrich Kauczor, Hyungseok Jang, Sam Sedaghat

**Affiliations:** 1 Department of Diagnostic and Interventional Radiology, University Hospital Heidelberg, 69120 Heidelberg, Germany; patricia.leutz@med.uni-heidelberg.de (P.L.-S.); viktoria.palm@med.uni-heidelberg.de (V.P.); rene.mathy@med.uni-heidelberg.de (R.M.M.); hans-ulrich.kauczor@med.uni-heidelberg.de (H.-U.K.); 2 German Cancer Research Center (DKFZ), 69120 Heidelberg, Germany; martin.groezinger@dkfz-heidelberg.de; 3 Department of Radiology, University of California Davis, Davis, CA 95616, USA; hyjang@ucdavis.edu

**Keywords:** large language models, chatbot, ChatGPT, Gemini, radiology, management

## Abstract

**Background/Objectives**: Despite the growing popularity of large language models (LLMs), there remains a notable lack of research examining their role in workplace management. This study aimed to address this gap by evaluating the performance of ChatGPT-3.5, ChatGPT-4.0, Gemini, and Gemini Advanced as famous LLMs in responding to workplace management questions specific to radiology. **Methods:** ChatGPT-3.5 and ChatGPT-4.0 (both OpenAI, San Francisco, CA, USA) and Gemini and Gemini Advanced (both Google Deep Mind, Mountain View, CA, USA) generated answers to 31 pre-selected questions on four different areas of workplace management in radiology: (1) patient management, (2) imaging and radiation management, (3) learning and personal development, and (4) administrative and department management. Two readers independently evaluated the answers provided by the LLM chatbots. Three 4-point scores were used to assess the quality of the responses: (1) overall quality score (OQS), (2) understandabilityscore (US), and (3) implementability score (IS). The mean quality score (MQS) was calculated from these three scores. **Results:** The overall inter-rater reliability (IRR) was good for Gemini Advanced (IRR 79%), Gemini (IRR 78%), and ChatGPT-3.5 (IRR 65%), and moderate for ChatGPT-4.0 (IRR 54%). The overall MQS averaged 3.36 (SD: 0.64) for ChatGPT-3.5, 3.75 (SD: 0.43) for ChatGPT-4.0, 3.29 (SD: 0.64) for Gemini, and 3.51 (SD: 0.53) for Gemini Advanced. The highest OQS, US, IS, and MQS were achieved by ChatGPT-4.0 in all categories, followed by Gemini Advanced. ChatGPT-4.0 was the most consistently superior performer and outperformed all other chatbots (*p* < 0.001–0.002). Gemini Advanced performed significantly better than Gemini (*p* = 0.003) and showed a non-significant trend toward outperforming ChatGPT-3.5 (*p* = 0.056). ChatGPT-4.0 provided superior answers in most cases compared with the other LLM chatbots. None of the answers provided by the chatbots were rated “insufficient”. **Conclusions:** All four LLM chatbots performed well on workplace management questions in radiology. ChatGPT-4.0 outperformed ChatGPT-3.5, Gemini, and Gemini Advanced. Our study revealed that LLMs have the potential to improve workplace management in radiology by assisting with various tasks, making these processes more efficient without requiring specialized management skills.

## 1. Introduction

The COVID-19 pandemic underscored the crucial role of artificial intelligence (AI) in healthcare and medical practice, bringing AI’s potential to the forefront [1,2]. With a growing global population and rising healthcare costs, there is an urgent need for transformative changes in the healthcare sector [3]. The integration of AI presents a significant opportunity to accelerate these changes by enhancing hospital efficiency and enabling more precise healthcare delivery [4]. One of the AI applications that has gained significant attention is ChatGPT, an AI chatbot launched in November 2022, which quickly became widely popular [5]. Individuals from various professions, age groups, and regions began utilizing ChatGPT, contributing to its rapid ascent in prominence. Following this, Google introduced its own AI chatbot, initially named Bard, which was later rebranded as Gemini. Today, these and other AI chatbots are broadly referred to as “large language models” (LLMs) [6]. LLM chatbots like ChatGPT and Gemini can respond to a wide range of queries and generate responses by drawing on vast resources available on the internet [7].

As the global population ages and the demand for medical services increases, the healthcare sector must undergo significant transformations, and radiology is no exception [4]. Effective management in radiology is crucial for maintaining departmental performance while ensuring high-quality care and patient safety. Radiologists are increasingly expected to develop management and leadership skills, regardless of whether they hold prominent leadership positions, as the radiology workplace presents numerous opportunities for applying management strategies to enhance patient care and optimize departmental operations [4,8,9,10,11,12,13]. Accordingly, specialized skills and behavioral competencies to effectively address workplace challenges, meet evolving patient expectations, manage rising demands for care, and ensure continuous improvement in patient care become increasingly necessary for radiologists [14,15,16]. However, many radiologists may lack these essential skills unless they pursue additional education through traditional routes, such as earning a master’s degree in economics or business administration [8,9,10,11,12,13]. Accessing valuable information on radiology management through traditional methods can be daunting, often hindered by the overwhelming volume of sources and the complexity of medical management literature [8,9,10,11,12,13]. This is where LLMs, such as ChatGPT and Gemini, could provide accessible and efficient management support in radiology by addressing routine workflow challenges. Their integration is particularly valuable in this context, where the demands of high-volume imaging workflows and multidisciplinary collaboration often exceed the capacity of traditional workforce strategies. Although integrating LLMs into workplace management in radiology holds promise, no studies have yet explored their application in this field.

Therefore, this study addressed the specific research question: How do large language models (LLMs), particularly ChatGPT-3.5, ChatGPT-4.0, Gemini, and Gemini Advanced, perform on basic workplace management questions, consecutively augmenting the management of the radiology workplace for “everyone” without specific management skills? By conducting a comparative analysis of their performance, this study explored how these LLMs can support and enhance radiology workflow management, ultimately improving departmental efficiency and patient care. To the best of our knowledge, no prior research has examined the role of LLMs in tackling radiology-specific management questions, making this study a unique and valuable contribution to the field.

## 2. Materials and Methods

### 2.1. Design and Categories of Questions

ChatGPT-3.5 and ChatGPT-4.0 (both OpenAI, San Francisco, CA, USA) and Gemini and Gemini Advanced (both Google, Mountain View, CA, USA) were tasked with answering 31 pre-determined questions about workplace management in radiology (Supplemental File S1). Four question groups were derived: (1) patient management (n = 9), (2) imaging and radiation management (n = 7), (3) learning and personal development (n = 7), and (4) administrative and department management (n = 8). First, a comprehensive screening of available online sources was conducted to gather various interview and management-related questions. This process included identifying questions relevant to workplace management in radiology from reputable sources and aligning them with the study’s objectives. Additionally, real-world scenarios from the authors’ institutions were analyzed to ensure the inclusion of practical, context-specific questions reflecting everyday challenges faced in radiology management. From this combined pool of questions, 31 were carefully selected based on their relevance, diversity, and potential to assess the capabilities of large language models (LLMs) in addressing workplace management tasks. This selection process ensured a robust and realistic evaluation framework for the study. The two LLMs (ChatGPT and Gemini) and their respective versions, were selected for this study as they represent two of the most widely used LLMs.

### 2.2. Evaluation and Scoring of Responses

The responses from the LLMs were evaluated for quality using a recently developed mean quality scoring system [12], which incorporates three key dimensions: (1) overall quality score (OQS), (2) understandability score (US), and (3) implementability score (IS). A mean quality score (MQS) was calculated to provide an aggregate assessment by averaging these three scores. Each dimension was rated on a 4-point Likert scale, where the values were defined as follows: 1—insufficient, 2—moderate, 3—good, and 4—very good.

**Overall quality score (OQS):** This dimension evaluated the answer’s completeness, accuracy, and relevance.
  ○ Insufficient (1): The answer is incomplete, incorrect, or fails to address the question adequately.
  ○ Moderate (2): The answer partially addresses the question but lacks key points or includes inaccuracies.
  ○ Good (3): The answer is accurate and covers most critical aspects, with only minor omissions.
  ○ Very good (4): The answer is thorough, accurate, and addresses all key points effectively.
**Understandability score (US):** This dimension assessed the response’s clarity, organization, and ease of understanding.
  ○ Insufficient (1): The answer is confusing, poorly organized, or difficult to comprehend.
  ○ Moderate (2): The answer is somewhat clear but lacks proper organization or contains language issues that affect readability.
  ○ Good (3): The answer is clear, well-structured, and easy to understand, with only minor issues.
  ○ Very good (4): The answer is exceptionally clear, well-organized, and easy to read.
**Implementability score (IS):** This dimension measured the practicality and feasibility of the recommendations provided.
  ○ Insufficient (1): Recommendations are impractical, unrealistic, or too vague to implement.
  ○ Moderate (2): Some recommendations are practical, but others are vague, unrealistic, or require significant refinement.
  ○ Good (3): Recommendations are practical and largely feasible, requiring only minor adjustments.
  ○ Very good (4): Recommendations are highly practical, feasible, and readily implementable.

To ensure a rigorous and unbiased evaluation, two senior radiologists with advanced expertise in radiology workplace management independently assessed the responses from ChatGPT-3.5, ChatGPT-4.0, Gemini, and Gemini Advanced. The readers also determined which LLM performed better for each question. In cases of disagreement, a consensus was required to establish the superior answer for a given response. This robust assessment process allowed for a comprehensive comparison of the chatbots’ performance and potential applicability in radiology workplace management. The scoring system was validated on several training questions on various topics before this study.

**Figure 1:**
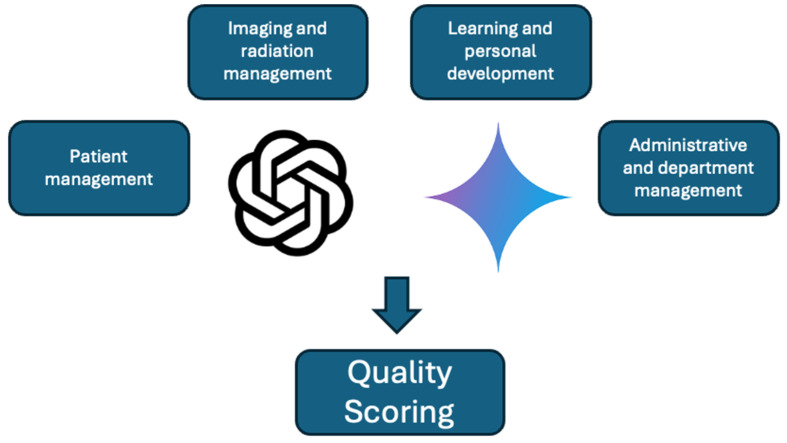
Flowchart of study design. Four topics on radiology workplace management were chosen, with several questions for each topic. ChatGPT-3.5, Gemini, ChatGPT-4.0, and Gemini Advanced generated responses to each question, which were analyzed by two readers using a novel quality scoring system.

Figure 1 presents the study concept.

### 2.3. Statistical Analysis Methods

Data were presented as mean values with standard deviation (SD), numerical counts, or percentages, depending on the nature of the variable. Descriptive statistics were employed to summarize and describe the data comprehensively. Inter-rater reliability (IRR) was assessed using Cohen’s Kappa. The IRR was categorized as moderate (41–60%), good (61–80%), or excellent (81–100%) based on standard interpretation criteria. The Mann–Whitney U-Test was applied to compare group values for the scores of each LLM chatbot. This non-parametric test was chosen because it does not require the assumption of normality and is suitable for ordinal or non-normally distributed data. Statistical significance was determined at a threshold of *p* < 0.05. All statistical analyses were conducted using IBM SPSS Statistics version 28.0 (IBM, Armonk, NY, USA). Python 3.12 (Python Software Foundation) was utilized to generate charts, summarizing the findings and highlighting differences between groups. The charts were designed to improve the clarity and interpretability of the results.

## 3. Results

### 3.1. Inter-Rater Reliability

The overall inter-rater reliability was good for Gemini Advanced (IRR 79%), Gemini (IRR 78%), and ChatGPT-3.5 (IRR 65%), and moderate for ChatGPT-4.0 (IRR 54%). Regarding the question groups, the IRR ranged from moderate (54%) to good (75%) for ChatGPT-3.5 (Group 1: 59%, Group 2: 54%, Group 3: 69%, and Group 4: 75%), from good (62%) to excellent (100%) for Gemini (Group 1: 75%, Group 2: 62%, Group 3: 72%, and Group 4: 100%), from moderate (58%) to good (70%) for ChatGPT-4.0 (Group 1: 76%, Group 2: 58%, Group 3: 62%, and Group 4: 70%), and from good (62%) to excellent (100%) for Gemini Advanced (Group 1: 62%, Group 2: 70%, Group 3: 81%, and Group 4: 100%).

### 3.2. OQS, US, IS, and MQS

The overall MQS for all questions, including quality, understandability, and implementability, averaged 3.36 (SD: 0.64) for ChatGPT-3.5, 3.75 (SD: 0.43) for ChatGPT-4.0, 3.29 (SD: 0.64) for Gemini, and 3.51 (SD: 0.53) for Gemini Advanced. The mean OQS for ChatGPT-3.5 was 3.29 (SD: 0.68), the mean US was 3.5 (SD: 0.53), and the mean IS was 3.29 (SD: 0.68). For Gemini, the mean OQS was 3.06 (SD: 0.69), the mean US was 3.5 (SD: 0.5), and the mean IS was 3.31 (SD: 0.64). The mean OQS for ChatGPT-4.0 was 3.81 (SD: 0.4), the mean US was 3.77 (SD: 0.42), and the mean IS was 3.68 (SD: 0.47). For Gemini Advanced, the mean OQS was 3.45 (SD: 0.53), the mean US was 3.63 (SD: 0.48), and the mean IS was 3.47 (SD: 0.56). The highest OQS, US, IS, and MQS were achieved by ChatGPT-4.0 in all categories, followed by Gemini Advanced. None of the answers received an “insufficient” rating.

Table 1 shows the scoring results from ChatGPT-3.5, Gemini, ChatGPT-4.0, and Gemini Advanced.

**Figure 2:**
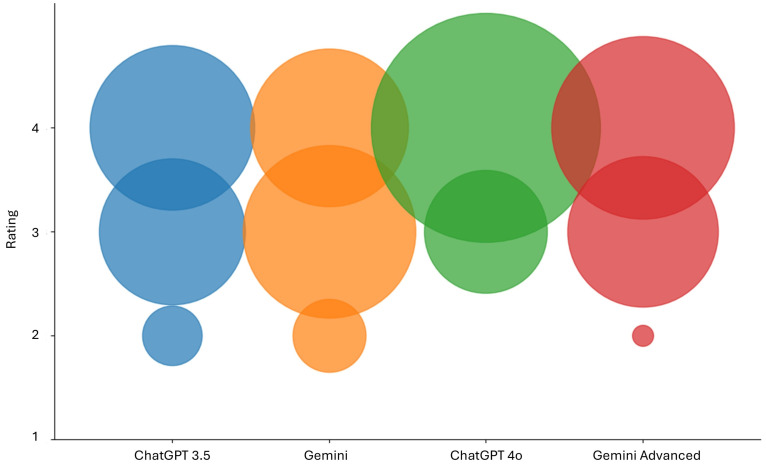
Bubble chart presenting the total number of ratings for answers provided by ChatGPT-3.5, Gemini, ChatGPT-4.0, and Gemini Advanced. The bubble sizes correlate with the rated scores for each chatbot.

Figure 2 shows the distribution of all performed ratings.

### 3.3. Significances

In thirty-four chatbot comparisons, significant differences in performance were observed among the LLM chatbots. Three analyses demonstrated a trend toward significance, though they did not reach statistical significance (*p* = 0.056–0.066). ChatGPT-4.0 emerged as the most consistently superior performer in most of the significant comparisons. Specifically, ChatGPT-4.0 significantly outperformed all other chatbots (*p* < 0.001–0.002). Gemini Advanced also performed significantly better than Gemini (*p* = 0.003) and showed a non-significant trend toward outperforming ChatGPT-3.5 (*p* = 0.056). The *p*-values for significant results ranged from <0.001 to 0.039.

Detailed significance analysis is provided in Table 2.

### 3.4. Superiority Analysis

**Figure 3:**
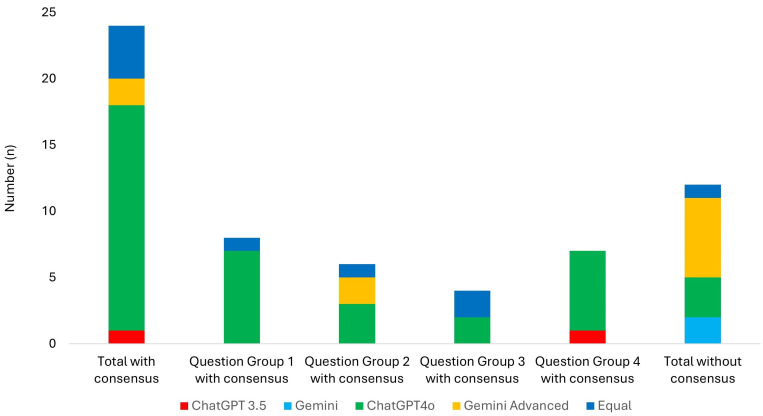
Results of the superiority analysis are organized into two main categories: findings with consensus of the two readers and findings without consensus. The findings with consensus are illustrated for each question group. In the findings without consensus, each chatbot’s mention is counted.

ChatGPT-4.0 provided superior answers in 17 cases (55%), where both readers reached a consensus. In comparison, the answers provided by Gemini Advanced were rated superior by consensus in two cases. ChatGPT-3.5 was rated superior by consensus in only one case. For Gemini, there was no consensus regarding superiority. Four answers were deemed equal across all LLM chatbots. In six cases, there was no consensus between the readers. The results of the superiority analysis are detailed in Figure 3.

## 4. Discussion

This study evaluated the potential of ChatGPT and Gemini, along with their two LLM versions (ChatGPT-3.5, ChatGPT-4.0, Gemini, and Gemini Advanced) in addressing radiology management questions across several key areas: patient management, imaging and radiation management, learning and personal development, and administrative and department management. All evaluated LLMs were found to be reliable for addressing radiology workplace management questions, with ChatGPT-4.0 performing the best overall, followed closely by Gemini Advanced. This approach ensured that the study assessed the LLMs’ performance under conditions that closely mirrored actual clinical and administrative contexts, thereby providing a realistic measure of their utility and applicability in real-world radiology workflows.

Our study utilized a recently introduced quality scoring system, offering a novel framework for evaluating the performance of LLMs [12]. According to this system, a score of 3 or higher signified a valuable and dependable resource. The scoring criteria focused on three critical aspects: the overall quality of the response, the clarity and depth of understanding demonstrated, and the ease with which the information could be applied in real-world scenarios. All the LLMs and their respective versions evaluated in this study were deemed reliable sources for addressing workplace management questions specific to radiology using that scoring system. ChatGPT-4.0 was identified as the most reliable and effective tool based on overall performance. It outperformed the other LLMs in most significance tests, followed closely by Gemini Advanced, indicating its superior ability to provide accurate and relevant responses to radiology-related management queries. However, it was noted that ChatGPT-4.0 exhibited the lowest inter-rater reliability (IRR) among the evaluated chatbots. This discrepancy may be attributed to the LLM’s tendency to generate lengthy and nuanced responses, which could have introduced variability in interpretation among raters. In contrast, Gemini Advanced demonstrated the highest IRR, likely due to its more concise and standardized answers. Nevertheless, the IRR reached only moderate to good levels, but not excellent values. Although the pure percentage agreement was relatively high (with a maximum disagreement of 20.5%), the IRR using Cohen’s Kappa also accounted for chance agreement, often resulting in lower values than the simple percentage agreement. Additionally, using only two readers may have been a further limitation, potentially influencing the IRR values and making the reliability assessment less robust. Future studies should include more readers to mitigate these limitations and enhance reliability.

For the evaluation, we selected 31 questions divided into four distinct categories, each meticulously designed to mimic real-world scenarios encountered in radiology practice. These scenarios encompassed common workplace challenges, decision-making dilemmas, and practical management issues that radiologists frequently face daily. With seven to nine questions per category, we believed that we could effectively capture and illustrate many aspects of daily workplace management in radiology. However, we acknowledge that there may still have been areas that were not fully addressed or covered in this study. Therefore, future studies should expand the number of questions and include a broader range of LLMs, as our study was limited to ChatGPT and Gemini.

Our study also revealed that as LLMs evolve, the quality of their responses tends to improve. This trend was particularly notable in ChatGPT, where the transition from earlier versions to more advanced ones markedly enhanced the quality of answers. In contrast, the improvement observed in Gemini was less pronounced, indicating a varying pace of development among different chatbot technologies. However, as there is still room for improvement in both LLMs, future developments will likely lead to enhanced performance of LLMs in the investigated field and beyond.

Guiding radiology departments through modern healthcare complexities, where clinical excellence and operational efficiency are paramount, becomes increasingly essential [17,18]. Accordingly, LLMs could streamline administrative tasks and optimize workflow within radiology departments. They could also serve as virtual assistants, providing quick access to critical information and resources. As these technologies evolve, their integration into radiology departments could lead to more organized, responsive, and patient-centered care environments. However, in the current literature, there is a noticeable lack of studies investigating the role of LLM chatbots in healthcare management, particularly within radiology. In radiology, existing studies have primarily focused on text-based research, particularly ChatGPT. For instance, a study by Chung et al. assessed ChatGPT’s ability to generate summarized MRI reports for patients with prostate cancer and evaluated physician satisfaction with these AI-generated summaries [19]. Similarly, Jiang et al. examined ChatGPT’s performance in transforming free-text radiology reports into structured reports for thyroid ultrasonography, finding that ChatGPT provided reasonably accurate results [20]. Schmidt et al. investigated the quality of LLM-simplified radiology reports as judged by medical professionals [21]. At the same time, Gordon et al. leveraged ChatGPT to generate patient-friendly recommendations based on imaging-related questions [22]. Hu et al. explored ChatGPT’s potential as an adjunct tool for generating diagnoses based on CT radiologic findings [23]. Our study is highly novel, as it paves the way for further exploration of LLMs beyond text-based analysis. Future research can build on our approach to examine the potential of LLMs in diverse radiological fields, such as workflow management and beyond.

While LLMs like ChatGPT and Gemini offer significant advantages, their use in radiology must be scrutinized from ethical and legal perspectives to ensure their integration does not compromise patient safety or professional accountability. Critical issues such as data privacy, security, and liability demand serious attention, as radiology workflows often involve processing sensitive medical information that must comply with stringent regulatory frameworks [24,25,26]. However, clear guidelines on the responsibility of radiologists when using LLM chatbots remain lacking, creating potential ambiguities in professional accountability when such tools are used for clinical decision-making or diagnostic assistance [25,26]. Moreover, current chatbots do not possess a genuine understanding of medicine or medical concepts; their responses are generated from training data that, while extensive, is not specifically tailored to address the nuanced and highly specialized demands of the medical field, including radiology [27]. The lack of specialized training in radiology-relevant data can lead to inaccuracies or misleading information, which, if unrecognized by the user, could negatively impact diagnostic accuracy and patient outcomes [19,28]. Furthermore, there is a risk of overconfidence in the outputs of these chatbots, as their highly fluent and confident responses might mask underlying inaccuracies, leading users to mistakenly believe the responses are always accurate or validated by evidence [29,30,31].

While LLMs hold the potential to serve as valuable advisors in radiology workplace management, they cannot substitute for the depth of knowledge and expertise gained through comprehensive management training and hands-on experience. These LLMs can provide insights, support decision-making, and enhance efficiency, but the nuanced understanding of complex organizational dynamics, interpersonal skills, and the ability to navigate unique workplace challenges are developed through real-world practice and experience. Therefore, while LLM chatbots can augment managerial efforts, they are most effective when used with the expertise of seasoned professionals in the field.

Our study showed that LLMs can serve as valuable tools for addressing workplace management challenges and providing guidance on tasks such as team coordination, patient management, and effective communication. By leveraging LLMs, radiologists may gain access to real-time recommendations and solutions without requiring extensive prior management training. This can lead to more targeted and efficient decision-making, allowing radiologists to focus on their clinical responsibilities while improving overall departmental workflow and collaboration. In the future, integrating LLMs into radiology practice could contribute to building a more dynamic and adaptive workplace where leadership and day-to-day management tasks are streamlined, ultimately enhancing operational efficiency and patient care quality. Accordingly, our study highlights the ability of these LLMs to deliver high-quality, actionable information that can support decision-making and workflow optimization.

This study had several limitations that should be acknowledged. First, we did not investigate variations in the chatbots’ responses, which could provide insights into their consistency and reliability. Future studies should explore how factors such as phrasing of questions, user input style, or repeated queries influence the responses generated by LLM chatbots. Additionally, we did not perform a systematic source verification or evaluate the depth and accuracy of the information provided by the chatbots. This limitation highlights the need for further research to validate the credibility and factual correctness of the outputs, particularly in high-stakes fields like radiology. Another limitation of this study lies in the evaluation methodology. While the mean quality scoring system provided a structured approach for assessing chatbot performance, it introduced potential biases due to the lack of a neutral option in the 4-point Likert scale. This could have influenced the evaluators to lean toward either favorable or unfavorable scores, potentially skewing the results. Future studies should consider incorporating a neutral option or exploring alternative scoring systems to minimize such biases. Furthermore, the generalizability of our findings is limited by the specific context of radiology workplace management. Although this study sheds light on the potential of LLMs in this domain, the applicability of these findings to other medical specialties or non-medical fields remains uncertain. Radiology presents unique challenges, such as high-volume imaging workflows, interdisciplinary collaboration, and the need for precise communication, which may not be directly comparable to other work environments. Future research should investigate the role of LLMs across a broader range of disciplines to determine how these tools can be generalized beyond radiology. Lastly, we aimed to address as many aspects as possible through our questions. However, there remain several areas that we were unable to cover fully.

## 5. Conclusions

ChatGPT and Gemini and their respective versions offer valuable guidance on radiology workplace management. ChatGPT-4.0 demonstrated superior performance, surpassing ChatGPT-3.5, Gemini, and Gemini Advanced, making it the preferred tool for radiology management tasks. However, while these LLM chatbots provide strong foundational support, they cannot substitute for the depth of sophisticated management experience and training that seasoned professionals bring to the table. The insights offered by these AI tools are beneficial, but they should be viewed as complementary resources rather than replacements for the nuanced understanding and decision-making capabilities that come from years of specialized training and real-world experience in radiology management.

## Figures and Tables

**Table 1:** Scoring results from ChatGPT-3.5, Gemini, ChatGPT-4.0, and Gemini Advanced regarding overall quality score (OQS), understandability score (US), and implementability score (IS). In addition, the mean quality score (MQS) is presented. Each question group (groups 1–4) is illustrated separately. Mean values with standard deviations (SDs) are shown.

Question Group	Mean ± SD			
	ChatGPT-3.5	Gemini	ChatGPT-4.0	Gemini Advanced
Overall Quality Score (OQS)				
1	2.88 ± 0.76	2.88 ± 0.77	3.77 ± 0.35	3.11 ± 0.46
2	3.07 ± 0.59	3.14 ± 0.52	3.79 ± 0.41	3.57 ± 0.5
3	3.17 ± 0.45	3.43 ± 0.5	3.79 ± 0.41	3.57 ± 0.5
4	3.56 ± 0.5	2.88 ± 0.78	3.88 ± 0.33	3.63 ± 0.48
Understandability Score (US)				
1	3.22 ± 0.48	3.33 ± 0.5	3.61 ± 0.48	3.44 ± 0.5
2	3.64 ± 0.48	3.64 ± 0.48	3.86 ± 0.35	3.79 ± 0.41
3	3.64 ± 0.48	3.57 ± 0.5	3.79 ± 0.41	3.57 ± 0.5
4	3.56 ± 0.5	3.5 ± 0.5	3.88 ± 0.33	3.75 ± 0.43
Implementability Score (IS)				
1	2.94 ± 0.7	3 ± 0.76	3.56 ± 0.5	3.16 ± 0.59
2	3.14 ± 0.74	3.43 ± 0.5	3.71 ± 0.45	3.57 ± 0.5
3	3.57 ± 0.5	3.5 ± 0.5	3.71 ± 0.45	3.57 ± 0.5
4	3.56 ± 0.5	3.38 ± 0.7	3.75 ± 0.43	3.63 ± 0.48
Mean Quality Score (MQS)				
1	3.02 ± 0.68	3.07 ± 0.66	3.65 ± 0.48	3.24 ± 0.54
2	3.29 ± 0.67	3.41 ± 0.54	3.79 ± 0.41	3.64 ± 0.48
3	3.64 ± 0.48	3.5 ± 0.5	3.76 ± 0.43	3.57 ± 0.5
4	3.56 ± 0.5	3.25 ± 0.72	3.83 ± 0.37	3.67 ± 0.47

**Table 2:** Results from the significance analysis. Significances were tested for the overall system, per scoring system, and per question group. “Chatbot 1” was tested against “Chatbot 2”. *p*-values < 0.05 were seen as significant.

Analyzed Groups	Chatbot 1	Chatbot 2	*p* Values
Overall	ChatGPT4.0	ChatGPT3.5	<0.001
	ChatGPT4.0	Gemini	<0.001
	ChatGPT4.0	Gemini Advanced	0.002
	Gemini Advanced	ChatGPT3.5	0.056
	Gemini Advanced	Gemini	0.003
Per Scoring System			
OQS	ChatGPT4.0	ChatGPT3.5	<0.001
	ChatGPT4.0	Gemini	<0.001
	ChatGPT4.0	Gemini Advanced	0.001
	Gemini Advanced	Gemini	0.005
US	ChatGPT4.0	ChatGPT3.5	0.012
	ChatGPT4.0	Gemini	0.009
IS	ChatGPT4.0	ChatGPT3.5	0.004
	ChatGPT4.0	Gemini	0.003
Per Question Group (1–4)			
MQS			
Question group 1	ChatGPT4.0	ChatGPT3.5	<0.001
	ChatGPT4.0	Gemini	<0.001
	ChatGPT4.0	Gemini Advanced	<0.001
Question group 2	ChatGPT4.0	ChatGPT3.5	0.001
	ChatGPT4.0	Gemini	0.004
	Gemini Adv	ChatGPT3.5	0.027
Question group 3	ChatGPT4.0	Gemini	0.039
Question group 4	ChatGPT4.0	ChatGPT3.5	0.023
	ChatGPT4.0	Gemini	<0.001
	Gemini Adv	Gemini	0.009
	ChatGPT3.5	Gemini	0.066
OQS			
Question group 1	ChatGPT4.0	ChatGPT3.5	0.001
	ChatGPT4.0	Gemini	0.001
	ChatGPT4.0	Gemini Adv	0.002
Question group 2	ChatGPT4.0	ChatGPT3.5	0.007
	ChatGPT4.0	Gemini	0.009
	Gemini Adv	ChatGPT3.5	0.063
Question group 4	ChatGPT3.5	Gemini	0.023
	ChatGPT4.0	Gemini	0.001
	Gemini	Gemini Advanced	0.014
IS			
Question group 1	ChatGPT4.0	ChatGPT3.5	0.019
	ChatGPT4.0	Gemini	0.027

## Data Availability

Data can be made available on reasonable request.

## Supplementary Materials

The following supporting information can be downloaded at: https://www.mdpi.com/article/10.3390/diagnostics15040497/s1, Supplemental File S1.

## References

[1] Pavli A., Theodoridou M., Maltezou H.C. (2021). Post-COVID Syndrome: Incidence, Clinical Spectrum, and Challenges for Primary Healthcare Professionals. Arch. Med. Res..

[2] Shaheen M.Y. (2021). Applications of Artificial Intelligence (AI) in healthcare: A review. Sci. Prepr..

[3] Thomson N.B., Rawson J.V., Slade C.P., Bledsoe M. (2016). Transformation and Transformational Leadership: A Review of the Current and Relevant Literature for Academic Radiologists. Acad. Radiol..

[4] Sedaghat S. (2024). The Future Role of Radiologists in the Artificial Intelligence-Driven Hospital. Ann. Biomed. Eng..

[5] Sedaghat S. (2023). Success Through Simplicity: What Other Artificial Intelligence Applications in Medicine Should Learn from History and ChatGPT. Ann. Biomed. Eng..

[6] Clusmann J., Kolbinger F.R., Muti H.S., Carrero Z.I., Eckardt J.-N., Laleh N.G., Löffler C.M.L., Schwarzkopf S.-C., Unger M., Veldhuizen G.P. (2023). The future landscape of large language models in medicine. Commun. Med..

[7] Sedaghat S. (2023). Early applications of ChatGPT in medical practice, education and research. Clin. Med..

[8] Lau L. (2007). Leadership and management in quality radiology. Biomed. Imaging Interv. J..

[9] Sedaghat S. (2024). Large Language Model-Based Chatbots Like ChatGPT for Accessing Basic Leadership Education in Radiology. Acad. Radiol..

[10] Buijs E., Maggioni E., Mazziotta F., Lega F., Carrafiello G. (2024). Clinical impact of AI in radiology department management: A systematic review. Radiol. Medica.

[11] Slanetz P.J., Omary R.A., Mainiero M.B., Deitte L.A. (2020). Educating the Next Generation of Leaders in Radiology. J. Am. Coll. Radiol..

[12] Leutz-Schmidt P., Grözinger M., Kauczor H.-U., Jang H., Sedaghat S. (2024). Performance of ChatGPT on basic healthcare leadership and management questions. Health Technol..

[13] Shipton H., Armstrong C., West M., Dawson J. (2008). The impact of leadership and quality climate on hospital performance. Int. J. Qual. Health Care.

[14] Abdi Z., Lega F., Ebeid N., Ravaghi H. (2022). Role of hospital leadership in combating the COVID-19 pandemic. Health Serv. Manag. Res..

[15] van Wart M. (2003). A comprehensive model of organizational leadership: The leadership action cycle. Int. J. Organ. Theory Behav..

[16] Daly J., Jackson D., Mannix J., Davidson P., Hutchinson M. (2014). The importance of clinical leadership in the hospital setting. J. Healthc. Leadersh..

[17] Mahoney M.C. (2020). Radiology Leadership in a Time of Crisis: A Chair’s Perspective. Acad. Radiol..

[18] Clements W. (2023). Understanding Leadership and its Vital Role in the Growth of Interventional Radiology. Cardiovasc. Interv. Radiol..

[19] Chung E.M., Zhang S.C., Nguyen A.T., Atkins K.M., Sandler H.M., Kamrava M. (2023). Feasibility and acceptability of ChatGPT generated radiology report summaries for cancer patients. Digit. Health.

[20] Jiang H., Xia S., Yang Y., Xu J., Hua Q., Mei Z., Hou Y., Wei M., Lai L., Li N. (2024). Transforming free-text radiology reports into structured reports using ChatGPT: A study on thyroid ultrasonography. Eur. J. Radiol..

[21] Schmidt S., Zimmerer A., Cucos T., Feucht M., Navas L. (2024). Simplifying radiologic reports with natural language processing: A novel approach using ChatGPT in enhancing patient understanding of MRI results. Arch. Orthop. Trauma Surg..

[22] Gordon E.B., Towbin A.J., Wingrove P., Shafique U., Haas B., Kitts A.B., Feldman J., Furlan A. (2024). Enhancing Patient Communication with Chat-GPT in Radiology: Evaluating the Efficacy and Readability of Answers to Common Imaging-Related Questions. J. Am. Coll. Radiol..

[23] Hu Y., Hu Z., Liu W., Gao A., Wen S., Liu S., Lin Z. (2024). Exploring the potential of ChatGPT as an adjunct for generating diagnosis based on chief complaint and cone beam CT radiologic findings. BMC Med. Inform. Decis. Mak..

[24] Sedaghat S. (2023). Future potential challenges of using large language models like ChatGPT in daily medical practice. J. Am. Coll. Radiol..

[25] Temperley H.C., O’Sullivan N.J., Mac Curtain B.M., Corr A., Meaney J.F., Kelly M.E., Brennan I. (2024). Current applications and future potential of ChatGPT in radiology: A systematic review. J. Med. Imaging Radiat. Oncol..

[26] Sedaghat S. (2024). Plagiarism and Wrong Content as Potential Challenges of Using Chatbots Like ChatGPT in Medical Research. J. Acad. Ethics.

[27] Ullah E., Parwani A., Baig M.M., Singh R. (2024). Challenges and barriers of using large language models (LLM) such as ChatGPT for diagnostic medicine with a focus on digital pathology—A recent scoping review. Diagn. Pathol..

[28] Shen Y., Heacock L., Elias J., Hentel K.D., Reig B., Shih G., Moy L. (2023). ChatGPT and Other Large Language Models Are Double-edged Swords. Radiology.

[29] Li H., Moon J.T., Iyer D., Balthazar P., Krupinski E.A., Bercu Z.L., Newsome J.M., Banerjee I., Gichoya J.W., Trivedi H.M. (2023). Decoding radiology reports: Potential application of OpenAI ChatGPT to enhance patient understanding of diagnostic reports. Clin. Imaging.

[30] Shahsavar Y., Choudhury A. (2023). User Intentions to Use ChatGPT for Self-Diagnosis and Health-Related Purposes: Cross-sectional Survey Study. JMIR Hum. Factors.

[31] D’antonoli T.A., Stanzione A., Bluethgen C., Vernuccio F., Ugga L., Klontzas M.E., Cuocolo R., Cannella R., Koçak B. (2024). Large language models in radiology: Fundamentals, applications, ethical considerations, risks, and future directions. Diagn. Interv. Radiol..

