# Supplementary material for: Performance of Large Language Models ChatGPT and Gemini on Workplace Management Questions in Radiology
Source: Diagnostics (Basel). 2025 Feb 19;15(4):497. doi: 10.3390/diagnostics15040497 (PMC11854386; doi:10.3390/diagnostics15040497)
Supplement: Supplementary file 1 [file diagnostics-15-00497-s001.zip › diagnostics-3355154-supplementary.pdf]

Supplemental material: questions

Patient management
How should emergency situations be handled during radiology examinations?
What steps can be taken to minimize patient discomfort during radiology procedures?
How can precision and accuracy be ensured during radiology examinations?
What measures should be taken to prevent infections after radiology procedures?
How should unexpected outcomes or complications arising from radiology procedures be addressed?
How can a patient's suitability for radiology procedures be evaluated?
What strategies can be employed to manage a difficult or non-compliant patient in a radiology setting?
How can patient confidentiality and privacy be effectively maintained within the radiology department?
What approaches can be used to manage patients who are anxious or uncooperative during radiology procedures?

Imaging and Radiation
How should I handle discrepancies between my interpretation of an image and that of another radiologist or clinician?
What steps to take to minimize radiation exposure for patients while still obtaining high-quality images?
What steps to take to minimize radiation exposure for patients while performing interventional procedures?
How to manage a difficult case where it is challenging to make a diagnosis?
How to communicate complex findings to referring physicians and other healthcare providers effectively?
What measures to take to protect myself and my team from radiation exposure?
What strategies should be used to ensure the accurate and timely reporting of results?

Learning and personal issues
How to stay up to date on the latest advancements in radiology technology and techniques?
What are the most important qualities for a successful radiologist to possess?
What is an effective approach to teaching and mentoring radiology residents and medical students?
What steps to take to stay current with continuing medical education requirements and professional development opportunities?
How to manage stress and maintain focus during long shifts or periods of high workload?
How to handle criticism or feedback from colleagues or superiors?

How to maintain strong relationships with referring physicians and other healthcare providers?

Administration and Department management

How to solve challenges in balancing clinical responsibilities with administrative tasks?
How to implement ethical considerations for radiologists?
How to ensure the efficient use of radiological equipment and resources?
How to approach quality improvement initiatives within my radiology unit?
How to adapt to new technology or protocols within my radiology unit?
How can I motivate my radiology department staff to work more productively?
How can I develop a vision for my radiology unit without overstraining my staff?
How can I measure the productivity of my radiology unit?
